# Differences in professional and interprofessional identity across years of professional education: a cross-sectional study of Norwegian health- and social care students

**DOI:** 10.1186/s12909-026-08893-6

**Published:** 2026-02-26

**Authors:** Ragna Stalsberg, Cathinka Thyness, Hilde Grimstad, Albertina Rusandu, Beate André, Randi Juul, Aslak Steinsbekk

**Affiliations:** 1 Faculty of Medicine and Health Sciences, Department of Circulation and Medical Imaging, Norwegian University of Science and Technology (NTNU), Trondheim, Norway; 2 Faculty of Medicine and Health Sciences, Department of Public Health and Nursing, Norwegian University of Science and Technology (NTNU), Trondheim, Norway; 3 Øya legesenter, Trondheim Municipality, Trondheim, Norway; 4 Faculty of Social and Educational Sciences, Department of Social Work, Norwegian University of Science and Technology (NTNU), Trondheim, Norway

**Keywords:** Profession, identity formation, interprofessional identity, higher education, health and social care, students, cross-sectional

## Abstract

**Background:**

There has been a call for studies investigating the assumption that professional and interprofessional identities evolve differently during educational programmes. The aim of this study was to investigate the level of professional and interprofessional identity across different years of health and social care students’ professional education.

**Methods:**

This cross-sectional study invited students across all study years from eleven health and social care higher education programmes at one university to complete a web-based questionnaire. Professional and interprofessional identity were assessed using the Macleod Clark Professional Identity Scale (MCPIS-9). Differences in identity scores between study years were examined using two-sample t-tests and multivariable regression models.

**Results:**

In total, 864 students, approximately one-fifth of the study population, participated. Across all study years, mean (SD) scores were 4.15 (0.54) for professional identity and 4.17 (0.53) for interprofessional identity (scale range 1–5). Final-year students scored slightly higher than first-year students on professional identity (mean difference 0.18, 95% CI 0.07 to 0.30), with a smaller difference observed for interprofessional identity (0.10, 95% CI − 0.01 to 0.21). The difference between first- and final-year students was 0.08 points larger for professional than for interprofessional identity. When stratified by programme length, the mean difference in professional identity between the first and final study year was 0.17 (*p* = 0.034) in three-year bachelor programmes and 0.30 (*p* = 0.022) in six-year professional programmes. Corresponding differences for interprofessional identity were smaller: 0.08 (*p* = 0.386) for bachelor programmes and 0.24 (*p* = 0.049) for six-year programmes.

**Conclusion:**

Students in later study years exhibited slightly higher professional identity scores than earlier years, with smaller between-year differences for interprofessional identity. The very small between-year differences may reflect lack of measurement sensitivity in MCPIS-9, stability in identity levels, high professional identity during the first year of study, or cohort effects inherent to cross-sectional designs.

## Background

Studies among single professions have shown that professional identity develops during professional education [1–3]. However, there is a paucity of studies exploring the level of students’ professional and interprofessional identities at different stages of their professional education in health and social care. Empirical research may increase our understanding of what is considered to be an under-theorised research field [4, 5].

Professional identity has been defined as ‘the attitudes, values, knowledge, beliefs and skills shared with others within a professional group and the professional role undertaken by the individual’ [6]. It is the outcome of integrating personal values, morals, and attributes with the profession’s norms [7, 8]. More generally, the concept is expressed as a type of social identity where members of a profession categorize and differentiate themselves from other professionals [9] and think, feel, and behave like members of their own profession [10]. It has been argued that professional identity formation requires enough time and space to ensure students achieve confidence in practising their profession [11, 12] and that a delay in professional identity formation potentially hampers a successful transition from student to practitioner [13].

In medical education, a stronger professional identity is associated with several factors, such as academic motivation [1] and higher preparedness for practice [14]. Furthermore, gender, previous work experience in health and social care services, understanding of teamwork, knowledge of the profession, and cognitive flexibility have been found to contribute to professional identity scores among first-year health and social care students [6] although later studies of paramedic students [2] and first-year German medical students [15] did not confirm these findings. The actual level of professional identities at different time points of higher education is, however, less studied. It could be expected that professional identity would be higher in the later years of the educational programme than at an early stage. However, only a slight difference has been identified in previous single-profession studies on Indonesian medical students [1], and in Australian paramedic students [2]. One review even found lower professional identity scores in the last years across studies of Chinese nursing students [3]. No cross-sectional studies on the level of professional identity in the different years among students from multiple health and social care educational programmes have been identified.

Nevertheless, it has been stated that a sound professional identity appears to develop in interprofessional learning situations where the students experience and discern their professional roles by interacting with other professional groups [16]. However, there is a still a paucity of research on interprofessional identity formation, and only limited studies have examined levels of interprofessional identity. Interprofessional identity formation has been defined as 'the development of a robust cognitive, psychological and emotional sense of belonging to an interprofessional community, necessary to achieve shared context-dependent goals' [17]. Thus, it is based on a broader circle of group membership consisting of more than one profession.

Interestingly, interprofessional identity formation is said to be a concept superordinate to professional identity [18, 19]. Underpinned by the interprofessional socialisation framework provided by Khalili et al. [20, 21], supported by qualitative findings by Woltenberg et al. [22], it has been suggested that interprofessional identity develops from professional identity, and thus depends on a prior identification with a specific profession.

Either way, educational institutions are responsible for nurturing both identities. Thus, knowledge about the level of professional and interprofessional identity during the students’ professional education can be helpful for designing apt curricula. Furthermore, previous reviews on professional [4] and interprofessional identity [5, 17] have called for studies investigating the assumption that professional and interprofessional identities evolve differently during educational programmes. Thus, there is a need for studies investigating both professional and interprofessional identity in the same students.

Hence, the overall aim was to investigate the level of professional identity and interprofessional identity at different stages of health and social care students’ professional education. This was done by answering the following research questions:

- RQ1. What are the scores on specific items, and the overall levels, of professional identity and interprofessional identity across all students and years?
- RQ2. Do professional identity and interprofessional identity scores differ between first, mid, and final year students?
- RQ3. When adjusting for student and parental characteristics, do professional identity and interprofessional identity scores differ between first, mid, and final year students?
- RQ4. When adjusting for student and parental characteristics, does the difference between professional identity and interprofessional identity scores differ between first, mid, and final year students?
- RQ5. Within three‑year bachelor programs and six‑year professional programs, do professional identity and interprofessional identity differ between study years?

### Methods

This was a cross-sectional study that collected data from health and social care students by means of a web-based questionnaire. The study was approved by the privacy officer (Norwegian Agency for Shared Services in Education and Research (SIKT), ref. 701327). All students received written information about the purpose of the study, how data was stored and protected, and that they consented to participate by submitting the questionnaire. Hence, all students who completed and submitted the questionnaire, consented to participate. The study was also approved by the department managements, including the relevant deans, to assure legitimacy of data collection among students, who tend to be overloaded with invitations to participate in research studies. The data were collected in the spring of 2022.

### Setting

The study was conducted at the Norwegian University of Science and Technology (NTNU), which has a total of 40,000 students across all types of programmes, and 13 different health- and social care study programmes. Two of these are six-year professional programmes (medicine and psychology), all the others are three years bachelor programmes (nursing, social care etc.). For most of these programmes, the grade requirements for admission from high school are the highest or among the highest in the country. In total, the health and social care study programmes have approximately 4,800 students. The learning outcomes for all these study programmes follow national regulations.

### Participants and recruitment

The study aimed to include students from all study years from all the health and social care study programmes at NTNU. Programmes with no graduates in 2021 or that did not offer the full programme leading to authorization, were excluded. This meant that two of the 13 study programmes were excluded: paramedic, as the study programme started enrolling students in 2021, and pharmacy, which is only offered as a two-year master’s programme for students who hold a bachelor from another institution.

To recruit participants, all eligible students were invited to participate by e-mail with a link to the questionnaire. Some students were provided information and time to complete the questionnaire in a break or immediately after a lecture. A reminder was sent within three weeks. The questionnaire was available for three months because students in different study programmes did not get the information at identical points in time, mainly due to the timing of their practice placements.

There are some differences in the amount of interprofessional educational activities across programmes. All 3rd year students from both the three-year bachelor programmes and the six-year professional study programmes participate in a two-day, on campus activity focusing on learning about other professions and coordination in health and social care on a system level. Additionally, all final-year students from these programmes participate in an eight-hours collaborative team-based practice placement centred on interprofessional assessment of a service user. Students from four specific programmes — audiology, bioengineering, psychology, and radiography students have a dedicated 7.5 credits (European Credit Transfer and Accumulation System) interprofessional longitudinal course which includes the two aforementioned activities (which also form a part of a joint 3rd year course of 15 ECTS credits for child welfare and social work students). Medicine and psychology students have a separate 7.5 ECTS credits interprofessional teamwork course, which includes all masters and six-year professional study programmes at NTNU including non-health programmes. Moreover, some study programmes have some minor additional interprofessional activities.

### Data collection and variables

The digital questionnaire was made available in the secure survey tool nettskjema.no, developed by the University of Oslo, Norway. The questionnaire consisted of questions about the participants and their parents/guardians, educational characteristics, and a battery of questions for assessment of professional and interprofessional identity.

Participant and educational characteristics included age, gender, study programme and year, whether the current study programme was their first choice among higher educational programmes in Norway, whether they had been studying at university level before the current programme, and whether they had worked in health and social care before or during their studies (yes/no). Moreover, questions about whether parents/guardians had been working in health and social care, and whether any of them had any tertiary level education (yes/no), were included in the questionnaire.

The length of study programmes was coded as three-year bachelor- and six-year professional study programmes. As a variable representing development during professional education, ‘academic progression’ was operationalised by recoding the variable ‘year of study’ (1st − 3rd year for the bachelor studies or 1st − 6th year for the six-year programmes of professional studies) into three categories: 1st year, midway (2nd year for the three-year bachelor studies, and 2^n^ − 5th year for the six-year programmes of professional studies), and final year.

Professional and interprofessional identity were assessed with the Macleod Clark Professional Identity Scale (MCPIS‑9) [6]. The instrument comprises nine items rated on a five‑point Likert scale (1 = strongly disagree to 5 = strongly agree). It was translated from English into Norwegian using a forward–backward procedure with three independent forward translations, consensus synthesis, and two independent back translations, followed by minor wording adjustments and final approval by the research group. For respondents with > 50% item completion, we calculated a mean score (range 1–5), with higher values indicating stronger identity. The scale was originally developed for health and social care students and has shown good psychometric properties, including a validated unidimensional structure and acceptable internal consistency in validation studies (Cronbach’s α of 0.78 and 0.79) [16, 23].

To allow for a comparable assessment of professional and interprofessional identity within the same measurement frame, we used the MCPIS-9 with the prompts 'this profession' and 'the health- and social care professions', respectively. This approach is consistent with the social‑identity–based conceptualizations underlying the MCPIS-9, which captures both professional and interprofessional identity as belonging to a salient in‑group characterized by shared attitudes, values, knowledge, beliefs, and skills as outlined in the introduction. Furthermore, preserving item content, response format, and directionality maintains the instrument’s inner logic and allows direct and interpretable comparisons.

### Sample size

The sample size for the larger study was calculated to have the power to detect a 10% difference between two groups, with a power of 0.9 and a statistical significance level of 0.05. To achieve this, 115 students in each group was required.

### Analyses

The characteristics of the whole sample is presented with frequencies, means and standard deviations. The same is done to present scores on each statement and identity score for subgroups. To compare professional and interprofessional identity, two-sample t-tests, presented with mean difference and 95% confidence intervals (95% CI), was used.

To analyse the development of professional and interprofessional identity during professional education, we built two multivariate regression models. Here, the dependent variables were professional identity and interprofessional identity; academic progression was the independent variable with adjustment for the variables gender, age, parents/guardians with higher education, parents/guardians working in health and social care, the student worked in health and social care before the study, and the student had study programme as first choice. The results of the multivariable analyses are presented with adjusted coefficients (adj. coeff.) and 95% CIs. The IBM Statistical Package for Social Sciences (SPSS) version 28.0.1.0 (142) was used for all analyses.

As there were only negligible differences between the development of professional and interprofessional identities for the whole sample and students on three-year bachelor’s and six-year professional study programmes separately, only a graphical presentation of the analysis of three-year bachelor’s and six-year professional study programmes is provided.

## Results

In sum, 864 students (approximately one-fifth of the study population) from 11 programmes completed the questionnaire. Their mean age was 23.9 years (standard deviation 4.1, median 23), and three in four were women (Table 1). One in five of the students were in their first year. Students in medicine, physiotherapy and nursing totalled two-thirds of all the respondents.

**Table 1:** Characteristics of responders. N (%) of the responders within each category. *N* = 864

Characteristics	*N* (%)
Academic progression	
- 1st year student	166 (19.2%)
- Midway student (2nd or 2nd -5th year)	416 (48.1%)
- Final-year student (3rd or 6th year)	278 (32.2%)
Gender	
- Women	668 (77.3%)
- Men	190 (22.7%)
- Non-binary/no answer	4 (0.2%)
Age group	
- < 21	219 (25.3%)
- 22–23	271 (31.4%)
- 24–25	184 (21.3%)
- > 25	186 (21.5%)
Parents/guardians	
- Have higher education	706 (81.7%)
- Work within H&S care	366 (42.4%)
The student	
- Worked in H&S care before studying	453 (52.4%)
- The study programme was the first choice	796 (92.1%)
Study programme	
- Audiology	1 (0.1%)
- Bioengineering	46 (5.3%)
- Child welfare	29 (3.4%)
- Medicine	315 (36.5%)
- Nursing	101 (11.7%)
- Occupational therapy	7 (0.8%)
- Physiotherapy	150 (17.4%)
- Psychology	43 (5.0%)
- Radiography	83 (9.6%)
- Social education	62 (7.2%)
- Social work	26 (3.0%)

### RQ1. Overall levels of professional identity and interprofessional identity

The variation in scores between the statements used to measure professional- and interprofessional identity was small, ranging from 3.6 to 4.6 on the 1–5 scale (Table 2). Similarly, there were only minor differences (maxim difference of 0.2) between each statement on the professional and interprofessional identity scale (Table 3). The mean scores across all statements were 4.15 and 4.17 for professional- and interprofessional identity, respectively.

**Table 2:** Scores for each statement and mean score across all statements for professional and interprofessional identity

Statements	Professional identity Mean (SD)	Interprofessional identity Mean (SD)	Difference Mean (SD)
I feel like I am a member of this profession/the H&S care professions	4.0 (0.79)	4.2 (0.73)	-0.19 (0.85)
I feel I have strong ties with members of this profession/H&S care professions	3.6 (0.96)	3.6 (0.90)	-0.02 (1.01)
I am often ashamed to admit that I am studying for this profession/ H&S care professions*	4.5 (0.82)	4.5 (0.73)	-0.08 (0.72)
I find myself making excuses for belonging to this profession/H&S care professions*	4.4 (0.97)	4.5 (0.75)	-0.18 (0.84)
I try to hide that I am studying to be part of this profession/ H&S care professions*	4.6 (0.77)	4.6 (0.64)	-0.07 (0.70)
I am pleased to belong to this profession/H&S care professions	4.4 (0.72)	4.3 (0.75)	0.10 (0.73)
I can identify positively with members of this profession/H&S care professions	4.3 (0.73)	4.1 (0.73)	0.15 (0.69)
Being a member of this profession is important to me/H&S care professions	3.9 (0.87)	3.8 (0.89)	0.05 (0.73)
I feel I share characteristics with other members of the profession/ H&S care professions	3.9 (0.86)	3.8 (0.80)	0.05 (0.75)
*Mean score across all statements*	*4.15 (0.54)*	*4.17 (0.53)*	*-0.02 (0.41)*

*Statements were phrased negatively but are reversed here to give all the statements the same direction

### RQ2. Professional identity and interprofessional identity by academic progression

There was a slight but marked difference in the students’ professional identity during professional education (0.19 points, *p* < 0.001) and a similar but weaker tendency for interprofessional identity (0.11 points, *p* = 0.075) (Table 3). There was a slight difference between the two identity scores, where the interprofessional identity score was highest, except for the final year, where the scores were similar.

**Table 3:** Professional and interprofessional identity scores with difference during professional education (academic progression)

Academic progression	*N*	Professional identity score (*N* = 860) Mean (95% CI)	Interprofessional identity score (*N* = 855) Mean (95% CI)	Difference (*N* = 855) Mean (95% CI)
1st year	166	4.04 (3.95 to 4.12)	4.10 (4.01 to 4.18)	-0.06 (-0.13 to 0.01)
Midway (2nd or 2nd -5th year)	411	4.13 (4.08 to 4.18)	4.16 (4.11 to 4.21)	-0.03 (-0.07 to 0.01)
Final year (3rd or 6th year)	278	4.23 (4.17 to 4.30)	4.21 (4.15 to 4.28)	0.02 (-0.03 to 0.07)

### RQ3. Adjusted professional identity and interprofessional identity by academic progression

Adjusted for other variables (gender, age, parents/guardians with higher education, parents/guardians working in health and social care, the student worked in health and social care before the study, and the student had study programme as first choice) the multivariate analyses gave similar results as the descriptive analysis. Last-year students had a mean professional identity score that was 0.18 (95% CI 0.07 to 0.30, *p* = 0.001) higher than the scores of 1st year students (Table 4), while the scores for interprofessional identity was 0.10 (95% CI -0.01 to 0.21, *p* = 0.075) higher (Table 5).

**Table 4:** Multivariate analysis of academic progression and professional identity adjusted for other variables

Characteristics	Professional identity		
	Mean score (SD)	Adj. coeff. (95% CI)	*P*-value
Academic progression			
- 1st year	4.04 (0.55)	REF	
- Midway (2nd or 2nd -5th year)	4.13 (0.53)	0.07 (-0.02 to 0.17)	0.138
- Last-year (3rd or 6th year)	4.23 (0.52)	0.18 (0.07 to 0.30)	0.001
Adjustment variables			
Women	4.14 (0.54)	-0.03 (-0.11 to 0.06)	0.538
Age group:			
- Age 21 years and younger	4.10 (0.50)	REF	
- Age 22–23	4.12 (0.53)	0.05 (-0.15 to 0.05)	0.314
- Age 24–25	4.17 (0.57)	-0.01 (-0.11 to 0.11)	0.932
- Age 26 years and older	4.20 (0.55)	-0.01 (-0.12 to 0,11)	0.906
Parents/guardians with higher education	4.13 (0.54)	-0.07 (-0.16 to 0.02)	0.149
Parents/guardians work in H&S care	4.15 (0.55)	0.02 (-0.06 to 0.09)	0.674
Student worked in H&S care before study	4.18 (0.51)	0.08 (0.00 to 0.15)	0.043
Student had study programme as 1st choice	4.17 (0.51)	0.36 (0.23 to 0.50)	< 0.001

**Table 5:** Multivariable analyses of academic progression and interprofessional identity adjusted for other variables

Characteristics	Interprofessional identity		
	Mean score (SD)	Adj. coeff. (95% CI)	*P*-value
Academic progression			
- 1st year	4.10 (0.56)	REF	
- Midway (2nd or 2nd -5th year)	4.16 (0.52)	0.05 (-0.05 to 0.15)	0.320
- Last year (3rd or 6th year)	4.21 (0.52)	0.10 (-0.01 to 0.21)	0.075
Adjustment variables			
Women	4.18 (0.52)	0.07 (-0.02 to 0.15)	0.127
Age group:			
- Age 21 years and younger	4.16 (0.49)	REF	
- Age 22–23	4.10 (0.54)	-0.09 (-0.17 to 0.01)	0.081
- Age 24–25	4.18 (0.54)	-0.01 (-0.12 to 0.10)	0.871
- Age 26 years and older	4.25 (0.55)	0.04 (-0.07 to 0.15)	0.491
Parents/guardians with higher education	4.15 (0.53)	-0.06 (-0.16 to 0.03)	0.183
Parents/guardians work in H&S care	4.18 (0.55)	0.03 (-0.05 to 0.10)	0.485
Student worked in H&S care before study	4.17 (0.53)	0.02 (-0.05 to 0.10)	0.538
Student had study programme as 1st choice	4.19 (0.51)	0.23 (0.10 to 0.36)	< 0.001

### RQ3. Adjusted professional identity and interprofessional identity differences by academic progression

The multivariable analysis showed that the professional identity score was 0.08 (95% CI 0.00–0.17, *p* = 0.058) higher than the interprofessional identity score when comparing all last year to all 1st year students (Table 6). Thus, the difference in students’ professional identity score from first to final year was 0.08 points larger than for the interprofessional identity score.

**Table 6:** Multivariable analysis of difference in professional and interprofessional identity

Characteristics	Difference between professional and interprofessional identity			
		Mean difference (SD)	Adj. coeff. (95% CI)	*P*-value
Academic progression				
- 1st year		-0.06 (0.45)	REF	
- Midway (2nd or 2nd -5th year)		-0.03 (0.40)	0.02 (-0.05 to 0.10)	0.568
- Last year (3rd or 6th year)		0.02 (0.39)	0.08 (0.00 to 0.17)	0.058
Adjustment variables				
Women		-0.04 (0.41)	-0.09 (-0.16 to -0.02)	0.008
Age group:				
- Age 21 years and younger		-0.04 (0.41)	REF	
- Age 22–23		0.01 (0.39)	0.04 (-0.04 to 0.11)	0.338
- Age 24–25		-0.01 (0.41)	0.01 (-0.07 to 0.10)	0.804
- Age 26 years and older		-0.05 (0.44)	-0.04 (-0.13 to 0.05)	0.336
Parents/guardians with higher education		-0.02 (0.42)	0.00 (-0.08 to 0.07)	0.916
Parents/guardians work in H&S care		-0.03 (0.39)	-0.01 (-0.07 to 0.05)	0.752
Student worked in H&S care before study		0.01 (0.40)	0.06 (0.00 to 0.11)	0.052
Student had study programme as 1st choice		-0.01 (0.39)	0.13 (0.03 to 0.23)	0.013

### RQ 5. Differences in professional identity and interprofessional identity by year within three and six-year programmes

**Fig. 1:**
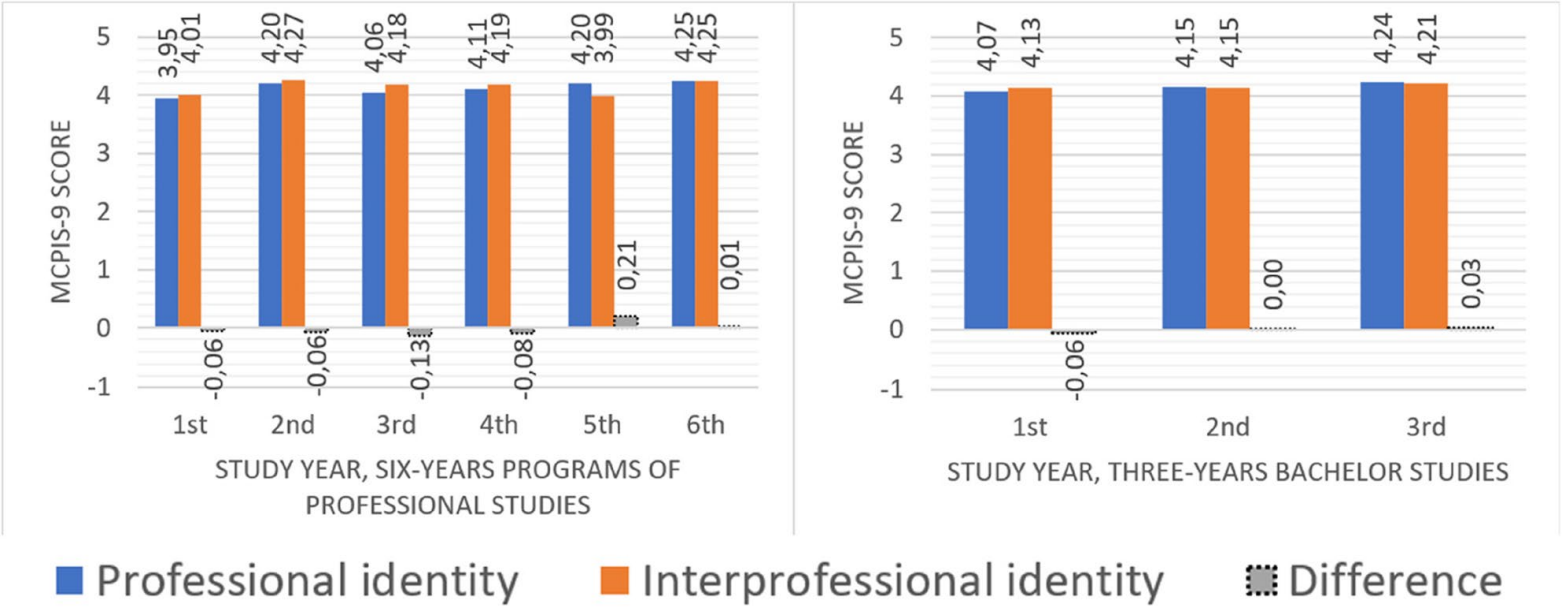
Mean professional and interprofessional identity scores by study years for the three- and six-year studies

As stated in the analysis, when dividing students in three-year bachelor- and six-year professional study programmes, there was no clear difference from the analysis of the whole group together. Therefore, only a graphical presentation of the differences between the two lengths of studies is provided (Fig. 1).

The difference in professional identity for students in three-year bachelor studies was 0.17 points higher in the last compared to the first year (*p* = 0.034) and for six-year professional study programmes the difference was 0.30 points (*p* = 0.022). However, this was less clear for interprofessional identity (0.08 points, *p* = 0.386 and 0.24 points, *p* = 0.049, respectively). For both bachelor students and students in the six-year professional study programmes, the interprofessional identity score was higher in the first part of their education, while the professional identity scores were higher at the end.

## Discussion

The professional- and interprofessional identity scores were almost the same among first-year students and was only slightly higher in the last year of professional education. The difference was somewhat larger for the professional than the interprofessional identity score. The same pattern was evident when looking at three-year bachelor- and the six-year professional study programmes separately. Thus, the overall finding was that health and social care students showed high levels of both professional and interprofessional identity from the first year with only minimally higher scores in the final year. The largest difference was observed for professional identity.

### Professional identity

The main finding indicates a small yet noticeable difference between students’ professional identity scores in the first and final year of their professional education. This was also evident when looking at students in the three-year bachelor- and the six-year professional study programmes separately.

Similar results have been reported in studies of professional identity in Indonesian medical students [1] and in Australian paramedic students [2]. Both studies applied the same MCPIS-9 scale as in the present study. These studies reported the MCPIS-9 score as the sum of the statements rather than the mean. Among the medical students, there was a difference in identity scores from the early to late study phases (35.8 to 40.7, *p* < 0.05), and among the paramedic students, a somewhat smaller difference was found between the first and last year of the study programme (37.8 to 40.7, *p* = 0.066). Studies of nursing students in China included in a review by Mao et al. [3] have reported a lower professional identity level among Senior students compared to Freshmen. However, restricted access to relevant Chinese journals renders the instruments used in professional identity assessments unknown.

The professional identity score was 4.0 on the 1–5 scale among first-year students in our study, which can be considered a high score. The high scores in first-year students might be due to these students having a sense of professional identity before they begin their studies. Adams et al. [6] and Roberts et al. [24] also found that health and social care students report a high level of professional identity when they start their academic professional education, supporting our interpretations. Generally, this could mean that professional identity formation might occur before or during the process when the students decide which study programme to enrol in, as suggested by Khalili [20]. As proposed by Mao et al. [3], it might also be that first-year students have an ideal image of the profession in which they get educated.

An alternative explanation to our and other studies’ findings of a relatively high professional identity among first-year students could be that the first-year students answered the questionnaire on professional identity in the spring term, i.e. after they had completed at least half to two-thirds of the first year. If they had completed the questionnaire immediately before or after the first day of their programme and reported a low professional identity, the observed high professional identity would be due to changes during the first part of the first year of studies. Such results would mean that students in their first year of study gain ample professional knowledge, values, and attitudes to identify with their profession almost as much as the soon-to-be graduates. Neither Johnston et al. [2] nor Mao et al. [3] have reported the timing of data collection from first-year students. Roberts et al. [24] collected professional identity data on first-year students at the end of the first semester and found professional identity scores equal to those of first-year students in the present study. These findings give reasons to call for further studies investigating professional identity development during the first year of study.

### Interprofessional and professional identity

The interprofessional identity scores were similar to the professional identity scores, and the topics raised in the discussion above about professional identity are also relevant to interprofessional identity. Thus, repeating them is redundant.

However, it is noteworthy that the students’ interprofessional identity score was slightly higher than their professional identity scores up to the end of the education. Based on the conceptualisation of Reiners [18] and the assumption embedded in the socialisation framework of Khalili et al. [20, 21] that interprofessional identity develops later than professional identity, it was reasonable to expect that interprofessional identity scores would be lower than professional identity scores among the first-year students and also show a larger difference between first and last year students. However, this was not the case even though all students in the present study, including the final year students, had attended several interprofessional education programmes, which have shown to affect the level of interprofessional identity [21].

An alternative interpretation might be that it is less complicated for students in their first year to ‘identify with a broader circle of group membership consisting of more than one profession’ [19] (i.e. interprofessional identity) than to be able ‘to categorize and differentiate themselves from other professionals’ [9] and ‘think, feel and behave like members of their profession’ [10] (i.e., professional identity). Such an idea would mean that new students have a higher sense of identity with the group of professions rather than the specific profession they have chosen to educate themselves into. Thus, rather than developing an interprofessional identity from a professional identity base, the interprofessional identity is the foundation from which the professional identity develops. Further studies are needed to clarify this.

The identity scale used, MCPIS-9, had not been validated for assessing interprofessional identity. However, only modest changes were made to the original statements, with the term *profession* replaced with *health- and social care professions*. One possible interpretation is that the insignificant differences in the scores between the two identities could be due to the students not noticing the difference. It could also mean that they consider their profession and the group of professions similar. Still, there were some differences, indicating that the respondents perceived the two versions differently.

As discussed above, the timing of the data collection may have influenced the interprofessional identity scores. Although first-year students are early in being socialized into the health and social care professions, their prior knowledge about these professional groups, in addition to the interprofessional learning programmes, may have shaped a level of interprofessional identity that makes it difficult to distinguish them from advanced students in terms of interprofessional identification.

### Strengths and limitations

The present study is the first cross-sectional study providing quantitative data on changes in both students professional and interprofessional identity during professional education. It is also the largest study, with students from all study years of eleven health and social care study programmes. The results are therefore important to increase the knowledge of health and social care students’ development of professional and interprofessional identity during professional education.

The response rate of one in five is a limitation, but similar or above other similar studies with response rates between 2 and 23% [23, 25, 26]. To assess representativeness, data about gender and age groups from the National Database for Higher Education Statistics (DBH) [27] about all the close to 5000 eligible students were retrieved. 81% were women and 65% were between 20 and 24. This aligns closely with the sample in this study, where there were 77% women and a median age of 23 with most in their early 20s. However, non-response bias cannot be ruled out, as respondents may differ from non-respondents in unmeasured ways, such as motivation, workload, or engagement with the study programme.

As this is a cross‑sectional study, the observed differences between study years may partly reflect cohort effects (i.e., systematic differences between the groups measured at one point in time rather than true change over time). For example, final‑year students may have entered with different characteristics than current first‑year students. However, the pattern of differences is small and fairly uniform, which makes a strong cohort‑effect explanation less likely. A longitudinal design following the same students over time is needed to confirm whether the year‑to‑year differences reflect within‑student development rather than between‑cohort variation.

The measurement used, the MCPIS-9 scale, has been validated and used in several studies. Still, it can be questioned if it is sensitive to change as only slight differences have been found between first and final-year students in all identified studies. The arguments for the measurement being sensitive to change despite studies finding only slight differences, would be that the students’ identity is stable during professional education. Thus, more research is needed, including the use or development of other measures. Furthermore, as both identity measures are based in self-report, social desirability bias and general response tendencies may have influenced scores and reduced differences between groups.

This cross-sectional study investigated the professional and interprofessional identity of different groups of students, which could mean that the observed differences could be due to a cohort effect. This means that students who were in their last year of study also would have scored higher on identity if they were asked as first year students compared to those who were in their first year when they participated. A longitudinal study that follows the same students during their whole education could thus yield different results and avoid the cohort effect.

Finally, this study was conducted at one university, which may have curricular structures and interprofessional programmes that differ from other universities. Generalisability to other contexts should therefore be made with caution.

## Conclusions

Across health and social care students from eleven different professional educational programmes, there was a slight yet noticeable difference between students’ professional identity at the start compared to the end of their professional education and a tendency for the same for interprofessional identity. The professional identity score showed a minor but larger difference during professional education compared to the interprofessional identity score. The very small size of the changes might be due to lack of sensitivity in MCPIS-9, or that students’ identity is stable during professional education. They can also be due to the students having a relatively high professional and interprofessional identity before starting a health or social care study programme. However, it might also be due to cohort effects as this was a cross sectional study.

## Data Availability

The datasets used and/or analysed during the current study are available from the corresponding author on reasonable request.

## Abbreviations

MCPIS-9: The Macleod Clark Professional Identity Scale
NTNU: Norwegian University of Science and Technology
ECTS: European Credit Transfer and Accumulation System
